# Bond Strength Survival of a Novel Calcium Phosphate-Enriched Orthodontic Self-Etching System after Various Ageing Protocols: An In Vitro Study

**DOI:** 10.1155/2022/3960362

**Published:** 2022-12-31

**Authors:** Noor M. H. Garma, Ali I. Ibrahim

**Affiliations:** ^1^Department of Orthodontics, College of Dentistry, University of Baghdad, Baghdad, Iraq; ^2^Centre for Oral Clinical and Translational Sciences, Faculty of Dentistry, Oral and Craniofacial Sciences, King's College London, London, UK

## Abstract

**Objective:**

This study aimed to evaluate the orthodontic bond strength and enamel-preserving ability of a hydroxyapatite nanoparticles-containingself-etch system following exposure to various ageing methods.

**Materials and Methods:**

Hydroxyapatite nanoparticles (nHAp) were incorporated into an orthodontic self-etch primer (SEP, Transbond™ plus) in three different concentrations (5%, 7%, and 9% wt) and tested versus the plain SEP (control) for shear bond strength (SBS), adhesive remnant index (ARI) scores, and enamel damage in range-finding experiments using premolar teeth. The best-performing formulation was further exposed to the following four artificial ageing methods: initial debonding, 24 h water storage, one-month water storage, and one-month acid challenge. A field-emission scanning electron microscope (FE-SEM) was used to examine the integrity of debonded enamel surfaces and calcium-phosphates (CaPs) reprecipitation.

**Results:**

The best-performing formulation (7% nHAp-SEP) resulted in significantly lower SBS (*p* < 0.001) than the control SEP following the four artificial ageing methods. Nevertheless, it survived the ageing protocols and yielded clinically acceptable SBS with the advantages of leaving minimal adhesive residue, preserving enamel integrity and smoothness, and inducing CaP reprecipitation as confirmed by FE-SEM images.

**Conclusions:**

A newly developed SEP produced adequate orthodontic bond strengths and left unblemished debonded enamel surfaces with minimal remnant adhesive and remineralisation potential, thereby suggesting simpler and safer bonding/debonding procedures.

## 1. Introduction

Direct bonding of orthodontic brackets using the conventional etch-and-rinse technique was first adopted by Newman [1]. Afterwards, numerous developments were launched to simplify orthodontic treatment and improve patient comfort. The technology of orthodontic bonding has taken a major leap onwards with the introduction of self-etch primers (SEPs), which represent simplified bonding systems as they circumvent the preliminary acid etching and after moist rinse control. Thus, they grant the advantages of low contamination risk and less technique sensitivity, more user-friendly, and can save chair-side time [2].

The bond strength of an orthodontic bonding system is an indispensable characteristic that has to be adequate to accomplish a successful orthodontic treatment. In addition, the optimal orthodontic bond strength is expected to preclude bracket failure throughout treatment whilst keeping the integrity of enamel at debonding after the course of orthodontic treatment [2]. Thus, a range between 5.9 and 7.8 MPa has been suggested as a clinically reliable orthodontic bond strength [3]. However, a higher range was recommended with a threshold value of 12 MPa, beyond which considerable enamel damage in the form of cracks or tear-out can occur. In general, SEPs have exhibited reliable performance with clinically acceptable bond strengths [2, 4]. However, there is a debate regarding the impact of SEPs on enamel's safety and the amount of adhesive remnants left after bracket debonding, as there is conflict about whether or not the amount of adhesive left is less or the same as the conventional technique [5, 6]. The adhesive remnants entail extra time and effort and impose inescapable enamel scratching at the post-debonding clean-up procedures [7]. Furthermore, enamel damage was seen after debonding of the brackets [4, 6, 8] and was reported to be more than the conventional etch-and-rinse technique [2, 9].

Biomimetic enamel remineralisation using synthetic hydroxyapatite nanoparticles (nHAp) has been widely studied in dental literature for their similar structure to the apatite crystals of human enamel. In addition, nHAp have excellent biocompatibility, low water solubility, robust adherence to enamel substrate and are considered an important provider of calcium and phosphate ions, which explain the capabilities of nHAp in reconstructing and remineralising the enamel [10]. Therefore, nHAp has been extensively studied and synthesized as biomimetic material in a wide size ranging between 50 and 1000 nm [10, 11]. An endeavour has been made to improve the remineralising ability of orthodontic bonding materials by incorporating nHAp into the resin adhesives [12] or bonding primers combined with other antimicrobial agents [13–15]. However, these attempts have merely used the conventional etching technique rather than the self-etch approach. In addition, they only partially addressed the clinical concerns, as their long-term ion release effectiveness, adhesive remnant reduction, and/or enamel damage prevention were not significant [16].

Therefore, this study aimed to develop an orthodontic bonding system combining the simplified SEP technique with nHAp remineralising properties and investigate the effect of nHAp incorporation on the enamel-bracket bond strength, enamel integrity, and the residual adhesive on the tooth surface. Thus, the following was hypothesised: (1) the enamel-bracket bond strength of the new orthodontic nHAp-SEP would be within the clinically acceptable range recommended in the literature; (2) the developed nHAp-SEP substantially decreases enamel damage and the residual adhesive on teeth upon bracket debonding compared with the plain SEP; and (3) the new bonding system can induce CaP reprecipitation.

## 2. Materials and Methods

This study was performed throughout three different phases.

### 2.1. Phase I: Range-Finding Pilot Experiments

#### 2.1.1. Development of nHAp-SEP Formulations

Orthodontic nHAp-SEP was developed using commercially supplied nHAp powder [Ca_5_(OH) (PO_4_)_3_] (Sigma-Aldrich, St Louis, MO, USA) of particle size <200 nm. Three different concentrations of 5% wt, 7% wt, and 9% wt were prepared in weight: weight ratios by adding the nanoparticles to the plain SEP; and the precise weight of nHAp and SEP was confirmed using a four-digit weight balance (KERN and SOHN GmbH, Germany). The plain single-step SEP (Transbond™ plus,3M Unitek, USA) was used as a control (TSEP), whilst each experimental SEP was prepared by adding the nHAp to a glass container containing the plain SEP, then mixed in an electric shaker for 20 s until a white homogenous suspension was obtained. All the formulations were separately and freshly prepared before use in the subsequent bonding procedure in a UV light-protected environment. A flat-surface pH electrode (S450CD, Sensorex, USA) was used to evaluate the pH values of the TSEP and the prepared formulations.

#### 2.1.2. Shear Bond Strength (SBS) and Adhesive Remnant Index (ARI) Assessment

Herein, after the ethical approval was obtained from the local ethics committee (Ref. number: 208), extracted human premolars were collected from 12–25-year-old orthodontic patients attending private or public Baghdad dental centres. Teeth selection criteria included an intact buccal enamel surface with no visible cracks, no caries or hypoplastic areas, and had not been chemically pretreated, as examined microscopically at x10 magnification. Then, the selected teeth were kept in a 1% chloramine-T trihydrate solution for 1 week and stored in distilled water (ISO/11405:2015) [17]. Subsequently, the premolars were mounted in acrylic blocks based on a standardised method [18]. Using simple random sampling, 40 mounted premolar teeth were assigned into four groups (*n* = 10) based on the SEP formulation used in brackets bonding: the control SEP (TSEP) and the three weight fractions (5%, 7%, and 9%) of nHAp referred to as 5nHAp-SEP, 7nHAp-SEP, and 9nHap-SEP, respectively.

Afterwards, teeth were polished with oil-free pumice (10 s), rinsed with water (10 s), and dried with compressed air (10 s). Then, the SEP material was applied with continuous rubbing on the middle third of the buccal surface for 5 s and lightly dried for 2 s. Each bracket base (stainless steel upper premolar brackets, Pinnacle, Orthotechnology, USA) was loaded with a thin layer of adhesive resin (Transbond XT, 3M Unitek, USA) and pressed onto the buccal surface for 3 s with a force gauge (Correx, Haag-Streit, Switzerland) using 300 g force and light-cured using a LED curing device (Elipar™ DeepCure-L, 3M ESPE, Germany) for 20 s (10 s for each proximal side) [2, 18]. Then, the bonded samples were kept in distilled water for 24 h at 37°C. The SBS test was accomplished using an Instron universal testing machine (Laryee WDW-50, Beijing, China); an occluso-gingival force was then applied by a chisel-like rod with 0.5 mm/min crosshead speed at the enamel-bracket interface until the bracket failed [18]. The recorded load (N) was divided by the surface area of the bracket base to compute the bond strength in MPa (1 MPa = 1 N/mm^2^). Subsequently, all the debonded buccal surfaces were coded and blindly examined under a stereomicroscope at x10 magnification. In addition, the residual adhesive was assessed based on ARI [19], which comprised a four-point scoring system: score “0, no residual adhesive left on the enamel surface; 1, less than half of the residual adhesive remained; 2, more than half of the residual adhesive remained; and 3, all adhesive remained with a distinct imprint of the bracket mesh.” Enamel damage in the form of a fracture or crack was examined at x10 magnification [4, 18]. Performance assessment of the different nano-SEP weight percentages was premised on the best outcomes regarding the SBS, with the least residual adhesive and enamel damage.

### 2.2. Phase II: Testing the New nHAp-SEP Formulation after Various Ageing Methods

A total of 160 extracted premolars were used in this phase. The best-performed SEP formulation obtained from phase I was further verified using a larger sample size with exposure to four separate models of artificial ageing. Firstly, the mounted premolar teeth were randomly allocated into two main groups (*n* = 80) based on the SEP formulation used for brackets bonding:

- Teeth bonded with the TSEP referred to as the control group
- Teeth bonded with the best-performed nHAp concentration referred to as nHAp-SEP

Then, specimens in each main group were further subdivided into four subgroups and separately subjected to four models of artificial ageing. Moreover, the criteria for teeth selection, mounting and bonding procedures, SBS, ARI, and enamel damage assessment followed in this phase were the same as those described in phase I.

#### 2.2.1. Artificial Ageing Models

- Initial debonding: the bracket debonding was carried out 30 min after bonding to simulate the intraoral archwire initial loading forces after bracket placement.
- Standard 24 h storage in water (24 HW): bonded teeth stored in distilled water (pH=6) for 24 h at 37°C before bracket debonding.
- One-month water storage (1 MW): the samples were kept in distilled water (pH=6) for 30 days at 37°C and the distilled water was replenished daily [20].
- One-month acid challenge (1 MA): the bonded teeth were stored in distilled water (pH = 6) for 24 h at 37°C before an acid attack protocol. The acidic challenge involved immersion of the samples in a 2.5 pH acidic solution of diluted hydrochloric acid (HCl) for a 5-minute session three times daily, separated by 2 h intervals, and stored in distilled water (pH = 6) for the rest of the day at 37°C to simulate the intraoral acidic effect on the bond durability. Subsequently, samples were rinsed under running water and air dried pre- and after each session. Both the acidic solution and the distilled water were replaced with a fresh solution after each session, and this protocol was repeated for 30 days [20]. The acidic solution was prepared by gradual dilution of concentrated (1 M) HCl in distilled water until the required pH was attained at ambient lab conditions. A digital pH meter (HI98103, HANNA, Romania) was used to determine the pH values for the distilled water and the acidic solution.

### 2.3. Phase III: Field-Emission Scanning Electron Microscope (FE-SEM) Examination of Enamel after Debonding of Brackets

Three teeth of each of the TSEP and nHAp-SEP from the 24 HW group were randomly selected for FE-SEM analysis. After bracket debonding, the crown of each selected premolar was sectioned mesiodistally through the occlusal central fossae using a metal abrasive disk under running water to obtain six buccal halves, which were sputter-coated with gold nanoparticles and examined with FE-SEM (Nova NanoSEM 450, FEI, Holland) at an accelerating voltage of 10 kV under low-vacuum operations. Finally, the debonded enamel surfaces were evaluated based on the enamel damage index [21], which includes the following categories:

  “Grade (0): smooth surface without scratches and perikymata might be visible
  Grade (1): acceptable surface with fine scattered scratches
  Grade (2): rough surface with numerous coarse scratches or slight grooves visible
  Grade (3): surface with coarse scratches, wide grooves, and enamel damage visible to the naked eye”

For better interpretation, the reader might use the abbreviation list in Table 1.

### 2.4. Statistical Methods

The sample size was calculated with a G-power version 3.1.7 (Franz Faul, Uni Kiel, Germany) depending on the comparison of SBS of different groups in the pilot experiment using one-way analysis of variance (ANOVA). Each subgroup needed a sample of 18 teeth to detect a significant difference with a large effect size at a 5% alpha level and 90% power. The SPSS software package (version 23, SPSS Inc., Chicago, USA) was used to conduct the statistical analyses at a level of significance of *p* < 0.05. In addition, data were screened for normal distribution and homogeneity using Shapiro-Wilk and Levene's variance tests, respectively. One-way ANOVA, Tukey HSD post hoc multicomparison and independent samples *t*-test were used for parametric data (SBS) with normal distribution and equal variances, whilst Kruskal–Wallis and Mann–Whitney tests were performed to compare the nonparametric data (ARI scores).

## 3. Results

### 3.1. Phase I: Pilot Study

The experimental SEP formulations 5nHAp-SEP, 7nHAp-SEP, and 9nHap-SEP presented higher pH values of 0.9, 1.3, and 1.9, respectively, as compared with the pH of TSEP (0.8). All parametric data exhibited a normal distribution with equal variances. The SBS outcomes of phase I experiments are shown in Table 2. The control group consistently displayed the highest SBS mean value, followed by lower values in the sequences 5nHAp-SEP and 7nHAp-SEP>9nHAp-SEP. The percentages of added nHAp exhibited significant effects on the bond strength based on the one-way ANOVA test among the three groups (*p* < 0.001). However, the significance of the drop in SBS was inconsistent when increasing the percentage of nHAp, as there was no significant difference between the 5% and 7% percentages, while those percentages exhibited significantly higher SBS than the 9% based on Tukey HSD post hoc multiple comparisons (*p* < 0.001).

The ARI scores and frequency of enamel damage are demonstrated in Table 3, which shows a significant effect of nHAp percentages on the amount of adhesive residue based on the Kruskal–Wallis test. The amount of adhesive that remained on the enamel surface of the control group was significantly higher (*p* < 0.05) than the nHAp groups based on the Mann–Whitney test, except for the 5nHAp-SEP group. Enamel conditioning with the TSEP and 5nHAp-SEP exhibited signs of enamel damage, whilst both 7nHAp-SEP and 9nHAp-SEP groups showed no enamel damage. A combination of the highest SBS with the least ARI and enamel damage was obtained from the 7nHAp, referred to as nHAp-SEP in the next sections.

### 3.2. Phase II

In phase II, the nHAp-SEP bond strength was tested with a larger sample size after various ageing methods. The bracket-enamel SBS results of this phase are shown in Table 4. Regardless of the SEP type, the SBS mean values decreased after 30-minute bracket debonding, whilst increasing after both 1 MW and 1 MA ageing methods as compared with the standard 24 HWS. However, these changes were statistically nonsignificant based on the one-way ANOVA test (*p* > 0.05). On the contrary, the control group yielded significantly higher SBS mean values than nHAp-SEP following the four ageing methods as indicated by independent samples *t*-test pairwise comparisons (*p* < 0.001).

The outcomes of ARI scores are shown in Table 5, which reveals nonsignificant differences among the different ageing methods for each SEP group based on the Kruskal–Wallis test. Conversely, analysis with Mann–Whitney demonstrated a significant decrease in the ARI scores between the SEP groups (*p* < 0.0001) for each ageing protocol. In addition, higher ARI scores (2 and 3) and enamel damage frequencies (13 enamel cracks and 5 fractures, i.e., 18 out of 80 samples) were only elicited in the plain SEP groups, whereas enamel surfaces treated with nHAp-SEP exhibited lower ARI scores (0 and 1) with clean, unblemished enamel.

### 3.3. Phase III: FE-SEM Examination

**Figure 1:**
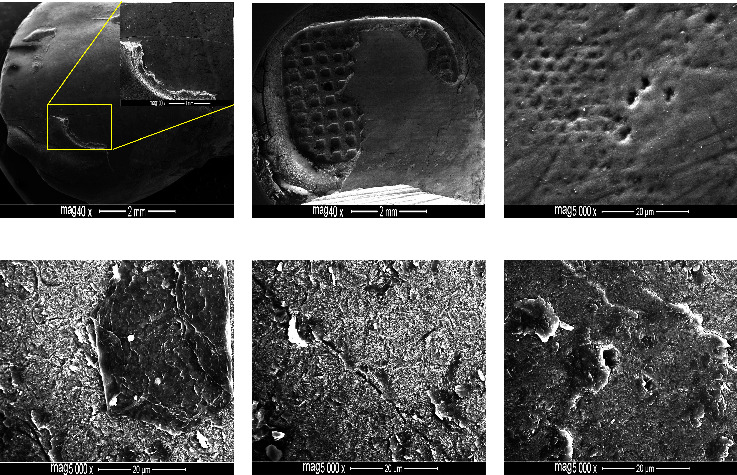
FE-SEM images of premolar buccal enamel surfaces etched with TSEP and debonded at 24 h. The etched teeth exhibited irregular, roughened enamel with abundant scratches, grooves, and cracks (a, e), adhesive residue (b, d), and open enamel micropores (c, f).

**Figure 2:**
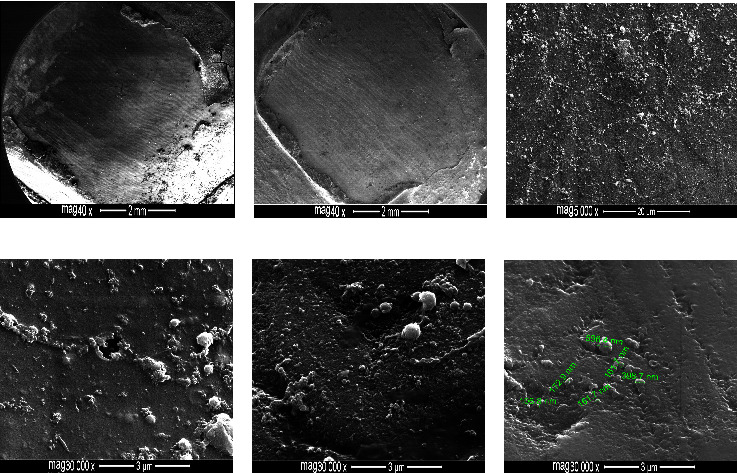
FE-SEM images of premolar buccal enamel surfaces etched with 7% nHA-SEP and debonded at 24 h. The conditioned teeth elicited regular, unblemished enamel surfaces with no or minimal residual adhesive (a, b) and abundant nCaP precipitations (c–f) abolishing and surrounding the micropores (d, e).

Enamel conditioning with the TSEP predominantly yielded cracked, roughened surfaces with abundant coarse scratches or grooving, identifying a combination of grades 2 and 3 with evident adhesive residues (Figures 1(a)–1(f)). In contrast, etching with nHAp-SEP yielded smooth surfaces with fine sparse scratches depicting a combination of grades 0 and 1 (Figures 2(a)–2(f)). In addition to maintaining immaculate enamel surfaces, no or minimal adhesive remnants with nCaP reprecipitation surrounding and blocking the enamel micropores (Figures 2(d) and 2(e)). Then, the nCaP precipitation was evident in the form of small granules or larger deposits (106.8–596.4 nm) (Figure 2(f)).

## 4. Discussion

The demand to simplify orthodontic techniques has led to the use of SEPs in orthodontic bonding, and the performance of conventional plain SEP has been investigated by various working groups [2, 4, 18]. Following these investigations, the bond strength values achieved with TSEP in this study were greater than the minimum range required for orthodontic clinical use. Hence, robust bonding of orthodontic attachments onto the enamel surface to move the teeth over an average duration of 1-2 yearswhich is pivotal for successful treatment. However, a correlation has been reported between bond strength and enamel damage incidence [2, 18]. The optimal SBS values in clinical orthodontics are expected to resist intraoral challenges during treatment yet permit safe brackets detachment after treatment, leaving as much intact enamel as possible. Therefore, the development of time-saving and enamel-preserving bonding systems aims to replace the traditional methods used for orthodontic bonding. The current study presented a newly developed SEP system with modified biomechanical properties by incorporating nHAp.

SEPs can be classified based on their acidic strength into strong (pH ≤ 1), intermediately strong (pH ≈ 1.5), mild (pH ≈ 2) and ultra-mild (pH ≥ 2.5); with an assumption that a deeper interaction with the dental substrate ensues as the acidity of the SEP rises [22, 23]. The bonding mechanism of TSEP (Transbond™ plus) was investigated and described as similar to that presented by the conventional etch-and-rinse technique, and this has been attributed to the etching aggressiveness produced by its strong acidity [23]. This one-step bonding system typically incorporates monomers such as methacrylated phosphoesters and 2-hydroxyethyl methacrylate (diHEMA) phosphate as functional monomers with water as a solvent. Herein, the calcium salts diHEMA-phosphate are formed after application to enamel; however, they are hydrolytically unstable and readily dissociate into pure phosphoric acid, so TSEP aggressively etches the enamel [22, 23]. The phosphate group dissolves and removes calcium and phosphate ions from hydroxyapatite (HAp), which in turn neutralises the acid and becomes incorporated into the network before the polymerisation of the primer. Meanwhile, SEPs have the advantage of complete infiltration into the demineralised area, thereby forming an intercrystallite nano-retention [2, 24]. This likely supports the high SBS achieved with these systems, yet adversely contributes to a higher risk of enamel damage during brackets debonding [2].

During the range-finding phase of this study, incorporating each of the three weight fractions of nHAp into the plain SEP significantly decreased the SBS, with a drastic drop when the weight fraction increased from 5% to 7% to 9%. Moreover, mixing nHAp powders with the plain SEP raised the pH value of the strong TSEP (pH=0.8), more evidently with the increase in the percentage of nHAp, hence alleviating the etching aggressiveness of the resultant nHAp-SEP. Incorporating nHAp into a system might elevate its pH value by releasing hydroxyl groups [12], and a comparable neutralising effect was reported in recent studies when adding calcium-containing compounds into phosphoric acid solutions [18] or SEPs [25]. The addition of nCaP as fillers results in structural reinforcement and enhances the adhesion of the resin material to some extent to the tooth substrate by exhibiting a primer-delivery carrier ability, thus improving the curing process [12] and strengthening the mechanical properties of the adhesive layer [13]. This limit was reported to be within the range of 0.1%–4% weight fraction of nCaP [12, 13, 26], beyond which an agglomeration or irregular dispersion of these nano-fillers might adversely influence the mechanical performance of bonding materials by forming defect points that interfere with the process of curing [13]. This may stand for the decrease in SBS associated with increasing the percentage of nCaP incorporated into the SEP. In addition, higher amounts of nano-fillers might increase primer viscosity, reduce wettability, and impede their flow into the micropores, which might contribute to lower bond strengths [26]. However, in this study, the 7nHAp-SEP showed comparable SBS mean values to the 5nHAp-SEP with significantly lower adhesive material and no visible enamel damage. Therefore, it was chosen as the best-performed formulation.

In addition to the standard 24 HW, three ageing methods were used to mimic the exposure of bonded interfaces to intraoral challenges and evaluate their effect on bond durability. During the initial orthodontic loading, early debonding was investigated to preclude the possible impact of inadequate bonding material homogeneity and incomplete polymerisation [27]. Similarly, bonding effectiveness might be affected by the hydrolytic degradation of water or the erosive capabilities of acidic drinks [28, 29]. There was a nonsignificant effect of the different ageing models on SBS for both the control and nHAp-SEP groups. The TSEP group survived all artificial ageing protocols recording the highest mean SBS values [27, 30]. However, bracket bonding with TSEP yielded significantly higher ARI scores with traumatic debonding after ageing (18 cracked or damaged enamel surfaces). Conversely, the incorporation of nHAp significantly reduced the SBS following all ageing methods, yet the mean values were within the clinically acceptable range reported in the literature (6–10 MPa) [2, 18]. Furthermore, the newly developed bioactive SEP (nHAp-SEP) interestingly yielded minimal or no residual adhesives after all debonding time points, including exposure to hydrolytic and acidic stresses. Moving the site of bracket failure towards the enamel-adhesive interface generally results in a reduced remnant adhesive amount with greater stresses and a higher risk of enamel damage during debonding [31]. Therefore, a bond failure may be preferred at the adhesive-bracket interface, but at the expense of increased chair time, cost, and effort to remove the remaining adhesive. In contrast, the developed nHAp-SEP was capable of moving the site of bond failure to the enamel-adhesive interface without jeopardising the integrity of enamel surfaces.

After debonding, the enamel surfaces were evaluated to assess the effect of nHAp incorporation into the TSEP on enamel integrity and potential nanomaterial interaction with the bonded surfaces, and FE-SEM was implemented instead of SEM to acquire precise and detailed structural information. The FE-SEM images exhibited superior performance of the nHAp-SEP in maintaining the integrity of debonded enamel surfaces, thus leaving a smooth, clean, unblemished substrate and interestingly outperforming the TSEP in preserving the perikymata with minimal or no residual adhesive. On larger magnification scales, the added nHAp reprecipitated and occluded the micro-pores either partially or completely. The reprecipitated clusters of nHAp obliterated the otherwise defected and grooved areas, thus creating smooth and mineralised surfaces as compared to the control's roughened, cracked, and open-micropores enamel substrate.

The nano-size of the added HAp can positively impact their performance by increasing the interfacial energy, hence playing a key role in their adsorption and interaction with the enamel surface. In addition, nHAp can outperform the microsized HAp in enhancing enamel remineralisation by increasing the bioavailability of mineralising ions (calcium and phosphate) under acidic pH conditions [32]. Therefore, the current study aimed to incorporate nHAp into a plain SEP to prevent the iatrogenic enamel damage associated with orthodontic bonding procedures at bonding/debonding time points. Hence, the recommended ion release to attain this goal was achieved during simultaneous enamel etching/priming. The added nHAp has high values of pH stability range (9.5–12) in aqueous solutions at 25°C, which are quite above the pH value of TSEP (pH=0.8). Thus, the strong TSEP can readily dissolute the incorporated nanoparticles [33], liberating calcium and phosphorus ions that enter the demineralised spaces and provide remineralisation seeds for HAp. These assumptions were confirmed by the FE-SEM images suggesting the enamel remineralising ability of nHAp-SEP with potential resistance to demineralisation during orthodontic treatment.

Similar to any *in vitro* study, the limitations in this study include variables related to the intraoral environment such as enamel composition, saliva contamination, or occlusal forces that significantly impact the bonding procedure and can alter *in vivo* outcomes. However, assessing bond strength following rigorous ageing methods was attempted to mitigate these variables. Hence, the presented *in vitro* model of orthodontic bracket bonding provides a preclinical and near-physiological basis to optimise the orthodontic adhesion process, with bracket bond strengths suitable for clinical performance. However, further testing is needed to assess the size and distribution of different wt% of nHAp within the TSEP for better insight into their different bonding behaviour. Hence, a randomised clinical trial is planned to evaluate the clinical efficacy of the developed nHAp-SEP system.

## 5. Conclusion

The newly developed orthodontic bioactive SEP with 7% nHAp yielded adequate bond strengths with less aggressive adhesion behaviour and induced enamel remineralisation through nCaP precipitation. In addition, the developed primer shifted the site of bond failure closer to the enamel surface. Hence, nHAp-SEP minimised adhesive remnants and enamel damage upon bracket removal, thereby enhancing the potential for reducing time, cost, and effort at the after treatment clean-up stage.

## Figures and Tables

**Table 1:** List of abbreviations used in this study.

Abbreviation	Description
nHAp	Hydroxyapatite nanoparticles
SEPs	Self-etch primers
TSEP	Transbond™ plus
CaP	Calcium-phosphates
SBS	Shear bond strength
ARI	Adhesive remnant index
nHAp-SEP	Best-performed hydroxyapatite nanoparticles-containing self-etch primer
5nHAp-SEP	5% hydroxyapatite nanoparticles-containing self-etch primer
7nHAp-SEP	7% hydroxyapatite nanoparticles-containing self-etch primer
9nHAp-SEP	9% hydroxyapatite nanoparticles-containing self-etch primer
24 HW	Standard 24 h storage in water
1 MW	One-month storage in water
1 MA	One-month acid challenge
FE-SEM	Field-emission scanning electron microscope

**Table 2:** Descriptive and inferential statistics of pilot shear bond strengths (SBSs) of bracket debonding at 24 h water storage.

SEP group	*N*	Min	Max	Mean SBS (MPa)	SD	Statistics: ANOVA	Statistics: Tukey HSD post hoc multiple comparisons
Control	10	10.7	24.1	18.1	4.7	*F* = 22.887 d*f* = 3 *p* < 0.001	*A * ^*∗*^
5nHAp-SEP	10	8.3	16.9	12.6	2.6		*B * ^*∗*^
7nHAp-SEP	10	9.8	16.3	12.6	2.3		*B * ^*∗*^
9nHAp-SEP	10	3.5	9.5	6.7	1.9		*C * ^*∗*^

The control group yielded significantly higher mean SBS values than the experimental nHAp formulations. (^*∗*^) Dissimilar letters indicate significant differences among groups at the 0.001 level.

**Table 3:** Descriptive and inferential statistics of pilot adhesive remnant index (ARI) scores of bracket debonding at 24 h water storage.

SEP group	*N*	ARI scores				Statistics: Kruskal–Wallis	Statistics: Mann–Whitney
		0	1	2	3		
Control	10	0	8 (2EC)	1 (1EC)	1	*H* = 22.189 d*f* = 3 *p* < 0.001	*A * ^*∗*^
5nHAp-SEP	10	6 (2EC)	4 (1EC)	0	0		*A*, *B*^*∗*^
7nHAp-SEP	10	7	3	0	0		*B * ^*∗*^
9nHAp-SEP	10	10	0	0	0		*B * ^*∗*^

The control group resulted in significantly higher scores than the experimental nHAp groups, except for 5nHAp-SEP. EC, enamel crack. (^*∗*^) Dissimilar letters indicate significant differences among groups at the 0.05 level. The significance values were adjusted by the Bonferroni correction for multiple tests.

**Table 4:** Descriptive and inferential statistics of shear bond strengths (SBSs) of control and nHA-SEP groups of bracket debonding at 24 h water storage (24 HW), 30 min (initial), one-month water storage (1 MW), and one-month acid challenge (1 MA).

SEP groups	Ageing method	*N*	Min	Max	Mean SBS (MPa)	SD	Statistics: ANOVA	Statistics: *t*-test (control versus nHAp-SEP)
Control	24 HW	20	10.73	25.88	18.49	4.49	*F* = 1.631 d*f* = 3 *p* > 0.05	24 HW^*∗*^, initial^*∗*^, 1 MW^*∗*^, 1 MA^*∗*^*p* < 0.001
	Initial	20	10.77	27.97	17.91	5.69		
	1 MW	20	12.74	26.97	19.77	4.06		
	1 MA	20	15.88	27.92	20.8	3.73		
nHAp-SEP	24 HW	20	9.76	17.85	13.46	2.67	*F* = 1.328 d*f* = 3 *p* > 0.05	
	Initial	20	9.42	17.67	12.87	2.66		
	1 MW	20	9.08	22.51	13.85	3.94		
	1 MA	20	9.11	19.16	14.85	3.47		

The control group produced significantly higher mean SBS values than the nHAp-SEP following all ageing methods. ^*∗*^ indicates significant differences between groups at the 0.001 level.

**Table 5:** Descriptive and inferential statistics of adhesive remnant index (ARI) scores of bracket debonding at four ageing protocols: 24 h water storage (24 HW); 30 min (initial), one-month water storage (1 MW), and one-month acid challenge (1 MA).

Groups	Ageing method	*N*	ARI scores				Kruskal–Wallis test	Mann–Whitney test (control versus nHAp-SEP)
			0	1	2	3		
Control	24 HW	20	1	14 (3EC+2 EF)	2 (1EC)	3	*H* = 6.803 d*f* = 3 *p* > 0.05	24HW^*∗*^, initial^*∗*^, 1MW^*∗*^, 1MA^*∗*^ *p* < 0.001
Control	Initial	20	1 (1EC)	14 (2EC)	4	1		
Control	1 MW	20	1 (1EF)	7 (2EC)	5 (1EC)	7		
Control	1 MA	20	1 (1EC)	10 (2EC+2EF)	4	5		
nHAp-SEP	24 HW	20	15	5	0	0	*H* = 4.086 d*f* = 3 *p* > 0.05	
nHAp-SEP	Initial	20	13	7	0	0		
nHAp-SEP	1 MW	20	17	3	0	0		
nHAp-SEP	1 MA	20	18	2	0	0		

The control group resulted in significantly more adhesive residue left on the enamel surface after all ageing methods. EC, enamel crack; EF, enamel fracture. ^*∗*^ indicates significant differences among groups at the 0.001 level.

## Data Availability

The data that support the findings of this study are available from the corresponding author upon request.
